# Supplementary material for: A versatile cortical pattern-forming circuit based on Rho, F-actin, Ect2, and RGA-3/4
Source: J Cell Biol. 2022 Jun 16;221(8):e202203017. doi: 10.1083/jcb.202203017 (PMC9206115; doi:10.1083/jcb.202203017)
Supplement: Table S1 — lists parameters. [file JCB_202203017_TableS1.docx]

Table S1. Model parameters

| **k_0_** | **0.00625 s^-1^** |
| --- | --- |
| **k_1_** | **0.3125 μM^-3^s^-1^** |
| **k_2_** | **1 μM^-2^** |
| **k_3_** | **0.0625 s^-1^** |
| **k_4_** | **0.05625 μM^-1^s^-1^** |
| **k_5_** | **0.0625 μM s^-1^** |
| **k_6_** | **0.02083 s^-1^** |
| **k_7_** | **0.001875 μM s^-1^** |
| **k_8_** | **0.140625 μM^-1^s^-1^** |
| **k_9_** | **0.25 μM^-2^** |
| **k_10_** | **0.025 s^-1^** |
| **D_RT_** | **0.08 μm^2^/s** |
| **D_RD_** | **0.4 μm^2^/s** |
| **D_F_** | **0-0.001 μm^2^/s** |
| **σ** | **0.5-1** |
| **s** | **4 μm** |
| **f** | **10-20 s** |
| **α** | **0.5-1.5** |
| **β** | **0-2** |
